# Three dimensional dual labelled DNA fluorescent in situ hybridization analysis in fixed tissue sections

**DOI:** 10.1016/j.mex.2014.04.001

**Published:** 2014-05-09

**Authors:** Kristin D. Kernohan, Nathalie G. Bérubé

**Affiliations:** a Departments of Biochemistry and Paediatrics, Western University, Victoria Research Laboratories, 800 Commissioners Road East, London, Canada; b Children's Health Research Institute, London, Canada; c Lawson Health Research Institute, London, Canada

**Keywords:** FISH, cryosections, tissue, fluorescence, confocal microscopy

## Abstract

Emerging studies demonstrate that three-dimensional organization of chromatin in the nucleus plays a vital role in regulating the genome. DNA fluorescent in situ hybridization (FISH) is a common molecular technique used to visualize the location of DNA sequences. The vast majority of DNA FISH studies are conducted on cultured cells due to the technical difficulties encountered using fixed tissue sections. However, the use of cultured cells poses important limitations that could yield misleading results, making in vivo analysis a far superior approach. Here we present a protocol for multiplexed three dimensional DNA FISH in mouse brain sections, which is also applicable to other tissues. Paraffin-embedded tissues could be used but the embedding and preparation of the samples is time-consuming and often associated with poor antigenicity. To overcome this problem we:

• developed a FISH technique using fixed, frozen cryosections;
• provide specific instructions for tissue processing for proper fixation and freezing, including equilibration in sucrose gradients to maintain proper cellular structure;
• include optimized permeabilization and washing steps to achieve specific signal and to limit background fluorescence in tissue sections.

## Graphical abstract

**Figure:**
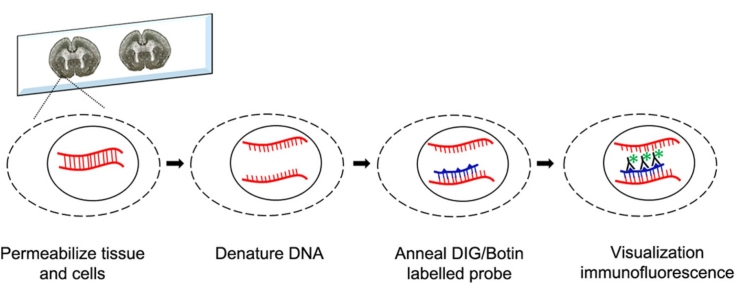

## Method details

### Step 1: tissue sample preparation

#### Materials

• Newborn mice
• Phosphate Buffered Saline (PBS) 4 °C
• 4% paraformaldehyde (PFA) in PBS
• 10% sucrose in PBS
• 20% sucrose in PBS
• 30% sucrose in PBS
• Cryomatrix (Fisher Scientific cat. 6769006)
• Plastic moulds (Fisher Scientific cat. 22038218)
• Dessicant
• Glass slides (VWR^®^ Superfrost^®^ Plus Micro Slide cat. 48311-703)

*Note*: This list includes only non-standard items. Common laboratory equipment such as liquid nitrogen is assumed to be available.

The choice of in vivo versus in vitro DNA FISH analysis is an important consideration in study design. In vitro analysis is complicated by several factors, including extensive molecular modifications as a result of growth in a non-physiological environment, and genetic and epigenetic changes required for transformation into an established cell line. Direct analysis of in vivo tissue circumvents these concerns. For in vivo analysis, proper fixation and preparation of the tissue are vital steps that require optimization to ensure experimental success. Paraffin-embedded tissues can be used for this analysis but the embedding and preparation of these samples is time-consuming and they tend to have poor antigenicity. We have developed a FISH technique using fixed, frozen cryosections. The preparation of cryosections involves less tissue manipulation while maintaining antigens for downsteam applications (e.g. combined DNA-FISH, with protein immunofluorescence). Sample preparation described herein utilizes neonatal brain tissue, but we have also tested this technique in other tissues (liver and thymus). For tissues that are more water saturated, such as the brain, equilibration in sucrose aids in maintaining cellular and tissue morphology. Optimization of the length of fixation may be required for tissues that differ substantially in size.

1. Neonatal mice were sacrificed by decapitation and the brains removed. Brains were placed into a petri dish with cold PBS on ice and rinsed twice in cold PBS.
2. Each brain was transferred to a 15 mL Falcon tube with 10 mL 4% PFA in PBS and left overnight to fix at 4 °C (14–19 h). In our experience it was best to dissect in the afternoon, and leave tissue in fixative until the following morning. Tissue fixation exceeding 24 h renders the tissue impermeable for subsequent hybridization and immunofluorescence reactions.
3. Fixative solution was decanted, and each brain was washed 3× 10 min with 10 mL cold PBS rocking gently at 4 °C. PBS was removed from samples.
4. 10 mL 10% sucrose in PBS was added and samples incubated at 4 °C for 24 h. At this point brains had sunk to the bottom of the tube. The sucrose solution was removed.
5. 10 mL 20% sucrose in PBS was added and the sample incubated at 4 °C for 24 h. At this point the brain had sunk to the bottom of the tube. The sucrose solution was removed.
6. 10 mL 30% sucrose in PBS was added and the sample incubated at 4 °C for 24 h. At this point the brain had sunk to the bottom of the tube. The sucrose solution was removed.
7. Brains were removed from 30% sucrose and placed on a Kimwipe to remove excess moisture and subsequently blotted dry with Kimwipe. Failure to properly dry the brain resulted in separation of brain tissue from cryomatrix during sectioning.
8. A thin layer of cryomatrix was placed in plastic moulds, and each brain was set into cryomatrix and remainder of the mould was filled until all the tissue was covered. Each sample was placed on a petri dish floating on the surface of liquid nitrogen to flash freeze. Samples were immediately stored at −80 °C.
9. Prior to sectioning, frozen brains were removed from −80 °C and placed in a cryostat (we use a LEICA CM 3050S but others are suitable) set at a temperature −20 °C for at least 30 min to equilibrate. Relevant brain regions were sectioned into 8 μM sections and placed on slides. In our experience thicker sections resulted in more background non-specific signal in downstream applications. Slides were left at room temperature overnight in an open slide box to dry. Dessicant packages were added and the boxes closed and stored at −80 °C.

### Step 2: probe preparation

#### Materials

• Bacterial Artificial Chromosome (BAC) DNA
• Biotin Nick Translation Mix (Roche diagnostics cat. number 11745824910)
• Digoxigenin (DIG) Nick Translation Mix (Roche diagnostics cat. number 11745808910)
• High pure PCR purification kit (Roche diagnostics cat. number 11732668001)

Immuno-FISH analysis requires highly specific probes and antibodies. After testing numerous types of labelled dNTPs (DNP, AMCA, FITC, biotin and DIG), antibodies, and probe preparation methods, we have determined that the following reagents and technical steps are optimal.

1. BAC DNA corresponding to the genomic region(s) of interest were obtained. Many genomics facilities provide extracted BAC DNA. It can also be extracted using the Qiagen Large Construct Kit (cat. number 12462). Alternatively, DNA sequences can be amplified by PCR from genomic DNA (as small as 2 kb FISH probes can be visualized by this method).
2. In a 1.5 mL tube, 1 μg of DNA was combined with 4 μL Roche Nick Translation Mix (either biotin or DIG) and H_2_O was added up to 20 μL. The solution was mixed gently by flicking.
3. Labelling reaction was placed in a 15 °C water bath for 90 min, then quenched by the addition of 1 μL 0.5 M EDTA pH 8.0 and incubation at 65 °C for 10 min.
4. Labelled probes were purified using Roche High Pure PCR purification kit according to manufacturer's instructions, with the following exceptions: after adding wash buffers, the columns were inverted 3 times prior to centrifugation. Similarly, after adding the elution buffer, columns were left to incubate for 10 min at room temperature. These extra steps increase the yield by 10–15%. Samples were eluted in a final volume of 100 μL elution buffer.

### Step 3: antigen retrieval

#### Materials

• 0.3% sodium citrate
• Coplin jar

**Fig. 1:**
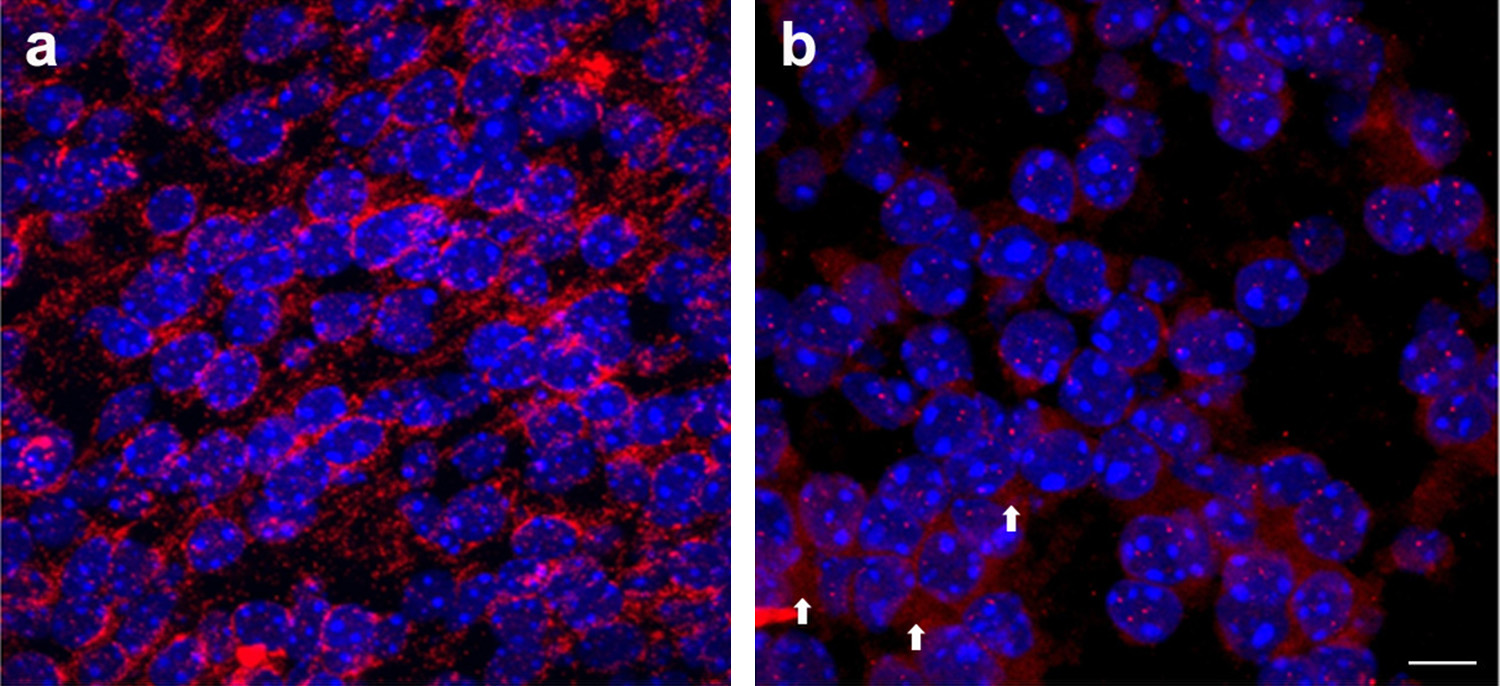
Examples of 3D FISH results obtained upon sub-optimal permeabilization or washing of the tissue sections (a) DNA FISH analysis of neonatal forebrain sections that were insufficiently permeabilized. Probe signal is seen in regions surrounding the cells but there is no specific signal in the nucleus. (b) Image of DNA FISH of neonatal brain sections that were not washed rigorously enough. In this case, a specific signal is observed in the nucleus, but is accompanied by a substantial amount of nonspecific signal. Examples of non-specific staining are indicated by white arrows. Scale bar: 6 μM.

This antigen retrieval step helps to permeabilize cells to reverse crosslinking of chromatin proteins but also increases DNA accessibility through DNA denaturation, resulting in increased probe access and probability of hybridization to its target sequence. This step is not required in standard cell culture assays. Insufficient permeabilization will result in probe being captured between cells, thus reducing signal in the nucleus (Fig. 1a). Antigen retrieval is conducted as follows:

1. Slides of brain sections were thawed in slide boxes from −80 °C to room temperature (this takes approximately 1–2 h). Slide boxes were not opened until they had reached room temperature to avoid condensation forming on sections, which would compromise their integrity.
2. A solution of 10 mM NaCitrate pH 6.0 was poured into a Coplin jar and heated to boiling in the microwave. The Coplin jar was removed from the microwave and left at room temperature until the solution ceased bubbling.
3. Slides were submerged in hot NaCitrate and the Coplin jar was placed back in the microwave and set on low for 10 min. The solution must be maintained at a temperature just below a simmer, as boiling would compromise the integrity of the tissue sections.
4. The Coplin jar was removed from the microwave and placed at room temperature until completely cooled (approximately 1 h).

### Step 4: fluorescent in situ hybridization

#### Materials

• 70% EtOH at 4 °C
• 90% EtOH at 4 °C
• 100% EtOH at 4 °C
• Hybridization buffer: 83% formamide, 3.3×SSC, 0.02 μM dextran sulfate, 20 μg salmon sperm DNA
• Rubber cement
• Glass cover slips
• Humidified chamber (damp paper towel in sealed plastic chamber)
• 37 °C incubator
• Denaturing solution: 70% formamide/2×SSC
• Wash buffer 1: 50% formamide/2×SSC
• Mouse anti-DIG antibody (Roche diagnostics cat. 11333062910)
• Rabbit anti-biotin antibody (Cell signalling cat. 5597)
• Goat anti-rabbit Alexa 594 antibody (Invitrogen cat. 11037)
• Goat anti-mouse Alexa 488 (Invitrogen cat. A-11001)
• Anti-Fade Gold (Invitrogen cat. S36936)

Denaturation and annealing of DNA probes is conducted as per standard FISH protocols (assay largely based on Zijlmans et al. [1]). However, the subsequent washing steps are performed for longer (10 min each) and with vigorous pipetting over samples to eliminate background inherent to FISH assays of tissue sections.

1. 2 μL of each probe was combined with 2 μL of 10 mg/mL salmon sperm DNA and 23.0 μL of hybridization buffer. Solution was mixed well by pipetting.
2. Probe was denatured by incubating at 65 °C for 10 min, then placed at 37 °C for 30–60 min (until slides were ready). During this time steps 3–5 were conducted.
3. Slides were removed from NaCitrate and dehydrated via an ethanol series: 70% EtOH for 2 min on ice, following by 90% EtOH for 2 min on ice and 100% for 5 min on ice. Slides were removed from EtOH and left on counter to dry (approximately 5 min).
4. To denature genomic DNA, slides were incubated in denaturing solution at 65 °C for 2 min.
5. Samples were dehydrated again via ethanol series: 70% EtOH for 2 min on ice, following by 90% EtOH for 2 min on ice and 100% for 5 min on ice. Slides were removed from EtOH and left on counter to dry (approximately 5 min).
6. 18 μL of the probe mix was added to the slide and covered with a glass coverslip, taking care not to introduce air bubbles. Coverslips were sealed to the slide with rubber cement and placed in a humidified chamber at 37 °C overnight (12–18 h). In our experience it is best to begin hybridization at the end of the day and process samples first thing the next morning. Incubation for longer periods of time (24+ h) significantly increases background levels.
7. Rubber cement was carefully removed with tweezers and slides placed in 45 °C wash buffer in Coplin jars. Tweezers were used to gently pull off coverslip while submersed in liquid. Slides were left to incubate in wash buffer for 10 min, while constantly pipetting solution over sections.
8. Tissue slides were washed a second time in 45 °C wash buffer for 10 min with continual pipetting over tissue sections. Insufficient washing results in probe accumulation between cells resulting in non-specific signal as depicted in Fig. 1b. This was eliminated by continuous pipetting of wash solutions over samples.
9. Sides were washed 3× 5 min in 0.3% TX-100/PBS at room temperature.
10. Primary antibody solution was added to detect the probes: anti-DIG used at 1:100 dilution and anti-biotin at 1:300 dilution in 0.3% TX-100/PBS. Samples were incubated for 1 h at room temperature.
11. Slides were washed 3× 5 min in 0.3% TX-100/PBS at room temperature.
12. Secondary antibody solution was added: goat anti-rabbit Alexa 594 (1:800) and goat anti-mouse Alexa 488 (1:800) in 0.3% TX-100/PBS. Samples were incubated for 45 min at room temperature in the dark.
13. Slides were washed 3× 5 min 0.3% TX-100/PBS at room temperature.
14. DAPI (10 μg/mL in PBS) was added at room temperature and incubated for 2 min. Slides were washed 3× 5 min in room temperature PBS.
15. Slides were mounted with 10 μL Anti-Fade Gold reagent (Invitrogen) and the coverslip sealed with nail polish. While multiple mounting reagents are available, in our experience Anti-Fade Gold kept the signal stronger for longer and produced better quality images.

### Step 5: analysis

**Fig. 2:**
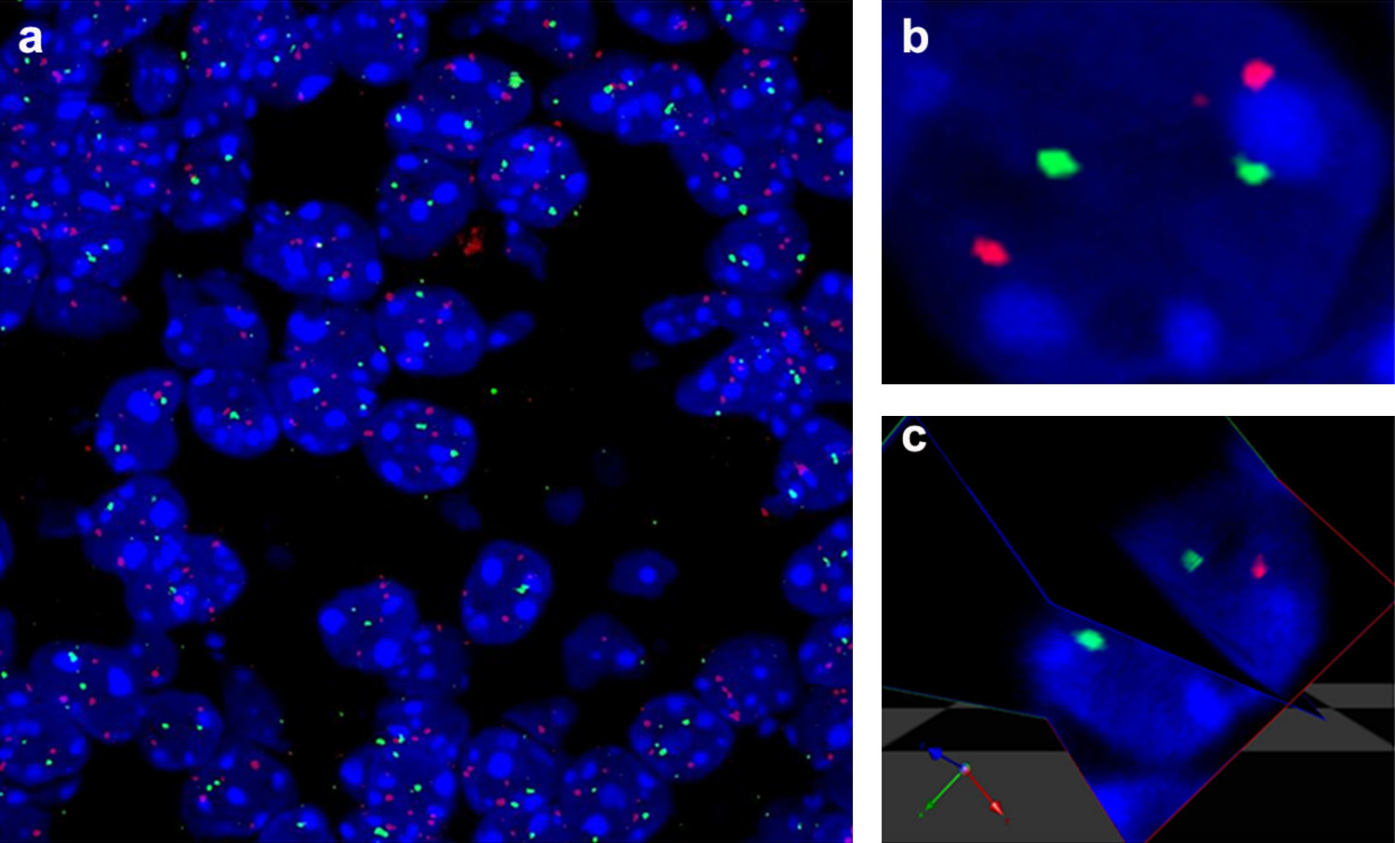
Confocal images of dual labelled DNA FISH in fixed mouse brain sections. (a) Example of field of view of neonatal mouse brain. Each cell has two foci per probe marking the two alleles. (b) Example of a collapsed image showing nuclear DNA foci and (c) its corresponding three dimensional rendering conducted with the Volocity software package. This data can be used to measure relative distances, signal volume or number of foci within the cell.

Slides can be kept at 4 °C for up to two weeks, but in our experience, the quality of the images decreased after 48 h. This length of time may vary according to the signal intensity, light intensity used and the type of microscope, and thus must be determined empirically. Z stack images were captured at high resolution in small intervals (0.3 μM) across the entire 8 μM section with a confocal microscrope and corresponding image acquisition software (we used the Olympics FV1000 confocal microscope and FV10-ASW 2.1 acquisition software). Volocity software was used for subsequent analyses (PerkinElmer), though other software packages are suitable. For localization and co-localization analysis, three dimensional rendering was imperative as collapsed images are not representative of true signal location. Additionally, multiple cells overlapped within the section and three dimensional analysis was necessary to assign FISH signals to their appropriate nuclear location. Using acquisition software, z stack images can be exported as .oif files and directly imported into Volocity. Image files were then visible in each plane or as a three dimensional representation (Fig. 2). Using the 3D measurement tools we measured distances in three dimensions by visualizing from different angles and planes. In designating signal localization or measuring distances between signals, the centre of the signal was always used. Centre to centre measurements ensure that differences in antibody accumulation between experiments do not affect the results. It is important to note that the thin sections used may not always contain complete cells. As such, care must be taken to use absolute measurements of distances within cells, and not use measurements proportional to cellular size.
